# Effects of Anticholinergic Drugs on Visual Acuity of Patients with Tracheal Intubation under General Anesthesia

**DOI:** 10.1155/2022/4559547

**Published:** 2022-06-20

**Authors:** Jinmei Song, Wenchao Shen, Yi Cheng, Yunhui Zheng

**Affiliations:** ^1^Department of Anesthesiology, Pinghu First People's Hospital, Pinghu, 314200 Zhejiang, China; ^2^Department of Pharmacy, Pinghu First People's Hospital, Pinghu, 314200 Zhejiang, China

## Abstract

**Background:**

General anesthesia (GA) is the core means of surgical intervention, mainly used for analgesia and anxiety relief. Therefore, it is necessary to understand the laboratory and clinical research results during induction of GA. Penehyclidine hydrochloride (PHCD) combined with atropine sulfate (Atr) has the potential to induce GA. However, the role of PHCD combined with Atr during tracheal intubation under GA remains unclear.

**Objective:**

The research is aimed at exploring the effects of preoperative PHCD or Atr on adverse reactions (ARs) in patients during tracheal intubation under general anesthesia (GA).

**Methods:**

This study retrospectively enrolled 473 patients who underwent surgery under GA induction and divided them into a research group (*n* = 234) and a control group (*n* = 239) according to preoperative use of PHCD (with or without). Both groups of patients were given Atr postoperatively and nursing intervention. Anesthesia-related indexes, ARs, and hemodynamics were observed and compared between the two groups.

**Results:**

There were no significant differences in anesthesia-related indexes and hemodynamics between the research group and the control group. The incidence of blurred vision and diplopia in the research group was higher than that in the control group.

**Conclusion:**

Preoperative PHCD combined with postoperative Atr should be avoided in clinical practice, or Atr rather than PHCD should be used preoperatively, so as to reduce the occurrence of blurred vision, diplopia, and other ARs.

## 1. Introduction

General anesthesia (GA), which is mainly used for analgesia and anxiety relief, is the core means of surgical intervention [1]. Although general anesthetics are widely used in surgical treatment, their mechanisms of action and potential side effects have not been fully clarified. In addition, these anesthetics may cause neurotoxicity to a certain extent, affecting brain development or causing cognitive impairment [2]. Kim et al. [3] pointed out that patients showed obvious hysterical somatic disorders during GA induced by propofol combined with lidocaine. GA will cause increased hemodilution due to infusion of crystalloid solution, leading to changes in plasma volume [4]. It can also affect hemodynamics, resulting in a series of perioperative complications, among which cardiopulmonary events are the most common type [5]. Besides, the kidney may play a vital part in the induction of GA, as follows: (1) kidney injury affects the excretion of anesthetic metabolites, and (2) the use of general anesthetics may lead to adverse renal outcomes [6]. In view of the hazards of the above-mentioned complications of GA to human health, it is necessary to understand the laboratory and clinical research results during induction of GA.

GA involves the initial induction by intravenous sedatives or analgesics and the subsequent maintenance by volatile anesthetics, as well as the postoperative use of antagonists decided based on the patient's condition [7]. Therefore, it is necessary to accurately understand the possible clinical outcomes that may be caused by the drug administration strategy during induction of GA. Atropine sulfate (Atr), a nonselective anticholinergic agent that can antagonize M1, M2, and M3 acetylcholine receptors, is mainly used to relieve smooth muscle spasms, inhibit glandular secretion, and relieve the inhibition of the vagus nerve on the heart. Yun et al. [8] believed that Atr could effectively prevent bradycardia caused by neostigmine during recovery from GA. Sahiner et al. [9] pointed out that Atr was safe in clinical application and can be used to increase patient comfort under GA. Penehyclidine hydrochloride (PHCD) is a novel selective anticholinergic agent with potent anticholinergic effects on both central and peripheral nerves, comprehensive and lasting. It is highly selective and antagonistic to M1 and M3 receptors but has no obvious effect on M2 receptor. Moreover, it can effectively antagonize organophosphorus poisoning symptoms, improve mucus secretion and vascular infiltration, and promote airway smooth muscle relaxation, allowing it to be an antidote to organophosphorus poisoning as well as a preanesthesia medication [10].

PHCD combined with Atr has the potential to induce GA. However, the role of the combination during GA induction remains unclear. In order to explore the effects of preoperative PHCD combined with postoperative Atr versus postoperative Atr plus postoperative Atr on adverse reactions (ARs) in patients with GA, we retrospectively enrolled 473 patients who underwent surgery under GA for analysis. The included patients were divided into a research group (*n* = 234) and a control group (*n* = 239) according to the preoperative use of PHCD (with or without), and the differences in anesthesia-related indexes, ARs, and hemodynamics were observed. The purpose of this study is to reveal the influence of PHCD plus Atr as well as postoperative Atr plus postoperative Atr on patients during induction of GA, thus providing a reliable research basis for clinical application.

## 2. Materials and Methods

### 2.1. General Information

The present study retrospectively analyzed the data of patients who received surgical treatment in Pinghu First People's Hospital under GA induction. Patients with anesthesia history, drug allergy history, severe chronic cardiopulmonary diseases, hepatic and renal insufficiency, cardiac insufficiency, arrhythmia, mental disorders, and alcohol addiction were excluded. After screening, 473 patients were included as research participants. This study was approved by the Ethics Committee of Pinghu First People's Hospital. In this study, patients were divided into a research group (*n* = 234) and a control group (*n* = 239) according to whether they were given PHCD preoperatively. In the research group, there were 125 (53.42) males and 109 (46.58) females, with an average age of 53.12 ± 15.97 years and an average body weight of 61.46 ± 11.17 kg. There were 53 (22.65) cases of American Society of Anesthesiologists (ASA) I, 121 (51.71) cases of ASA II, and 60 (25.64) cases of ASA III. The control group consisted of 121 (50.63) males and 118 (49.37) females, with a mean age of 53.02 ± 16.65 years and an average weight of 60.02 ± 13.83 kg. There were 62 (25.94), 126 (52.72), and 51 (21.34) cases of ASA I, II, and III, respectively. There was no statistical difference in age, gender, body weight, ASA score, and other general data between the two groups (Table 1), indicating that the two groups were feasible for subsequent research.

### 2.2. Administration Regimens

The administration regimen of the research group is as follows: 0.005-0.02 mg/kg PHCD (Jinzhou Avanc Pharmaceutical Co., Ltd., SFDA Approval No. H20020606) was injected intravenously 10 minutes before anesthesia induction to reduce airway secretion. The administration regimen of the control group is as follows: 0.5 mg Atr (Tianjin Jinyao Pharmaceutical Co., Ltd., SFDA Approval No. H12020382) was intravenously injected 10 minutes before anesthesia induction. The vital signs of the patient such as noninvasive blood pressure, electrocardiogram, and pulse oxygen saturation were monitored in real time after the patient entered the operating room. Induction plan is as follows: anesthesia induction was performed by intravenous injection of midazolam (0.05 mg/kg), vecuronium (0.8 mg/kg), and propofol (2.0 mg/kg). The patient was intubated 3 minutes after induction. Mechanical ventilation was performed after ensuring correct placement of the patient, with a tidal volume of 8-10 mL/kg, a respiration rate of 12-16 times/min, and a respiratory ratio of 1 : 2.

### 2.3. Nursing Intervention

Nursing intervention was carried out from psychological, cognitive, and emotional aspects. Psychological intervention is as follows: the communication between doctors and patients and their families was strengthened before operation, so as to understand the psychological state of patients. In addition, preoperative psychological counseling was given to patients to obtain their trust. Cognitive intervention is as follows: patients and their families were introduced to the basic process of surgery and anesthesia methods, so that patients could understand where they needed to cooperate. Emotional intervention is as follows: the relationship between emotions and diseases was explained from the perspective of biological-psychological-social medical model, and the negative effects of preoperative anxiety, tension, and other negative emotions on the operation were told to the patients' families to obtain their cooperation, all in an attempt to help patients eliminate the negative emotions.

### 2.4. Outcome Measures

1. Differences in anesthesia-related indexes, including anesthesia induction time, pain disappearance time, and recovery time, were observed in both groups
2. Differences in ARs (blurred vision, nausea, vomiting, and pain) were observed
3. The hemodynamic indexes, including heart rate (HR), mean arterial pressure (MAP), and blood oxygen saturation (SpO_2_), were also observed and compared between the two groups

### 2.5. Statistical Analysis

In this study, the measurement data is recorded as mean ± standard deviation, and the counting data is expressed as the number of cases (percentage). The data were tested for normality using the Shapiro-Wilk test. Independent samples *t*-test was used to compare the differences of anesthesia-related indexes and hemodynamics between the two groups. A chi-square test was utilized in the comparison of counting date such as ARs between the two groups. When *P* < 0.05 (95% confidence interval), the difference was considered statistically significant. All comparisons in this study were two-tailed.

## 3. Results

### 3.1. Effects of Preoperative PHCD Combined with Postoperative Antagonist Atr on Anesthesia-Related Indexes

To determine the role of PHCD combined with Art in GA, we analyzed the anesthesia induction time, pain disappearance time, and recovery time of patients in both groups, and the results are shown in Table 2. In the research group, the time of anesthesia induction, pain disappearance, and recovery was 4.14 ± 1.19 min, 3.81 ± 0.68 min, and 11.47 ± 2.01 min, respectively, while the data in the control group was 4.20 ± 1.10 min, 3.72 ± 0.61 min, and 11.53 ± 1.71 min, respectively. The statistical comparison revealed no significant difference in anesthesia induction time, pain disappearance time, and recovery time between the two groups (*P* > 0.05).

### 3.2. Effects of Preoperative PHCD Combined with Postoperative Antagonist Atr on Postoperative ARs

To determine the safety of PHCH combined with Atr during GA, we counted and compared the ARs between the research group (*n* = 234) and the control group (*n* = 239), with the results presented in Table 3. The number of patients with blurred vision, pain, nausea, and vomiting in the research group was 25, 82, 26, and 7, respectively, while in the control group, there were 84 cases of pain, 31 cases of nausea, and 15 cases of vomiting, without blurred vision. The above data revealed a significantly higher incidence of blurred vision in the research group compared with the control group (*P* < 0.0001) and no statistical difference in other adverse events between the two groups (*P* > 0.05), which indicated that preoperative PHCD combined with postoperative Atr resulted in postoperative blurred vision and diplopia in patients.

### 3.3. Effects of Preoperative PHCD Combined with Postoperative Antagonist Atr on Hemodynamics of Patients

To further determine the safety of PHCH combined with Atr in vital signs during GA, we statistically analyzed the hemodynamics of the two groups after recovery from anesthesia, and the results are shown in Table 4. The HR, MAP, and SpO_2_ in the research group were 85.48 ± 12.16 beats/min, 83.25 ± 10.01 mmHg, and 99.63 ± 1.31%, respectively, while in the control group, they were 85.34 ± 10.97 beats/min, 81.76 ± 11.36 mmHg, and 99.64 ± 0.88%, respectively. After statistical analysis, we found no statistical difference in the above hemodynamic indexes between the two groups after recovery from anesthesia (*P* > 0.05).

## 4. Discussion

Administration strategies during induction of GA should be cautious. In this study, we retrospectively analyzed 473 patients with surgical treatment under GA and compared the effects of different administration strategies implemented in the research group and the control group on ARs. The results showed that compared with preoperative and postoperative use of Atr, PHCD combined with Atr has no significant difference in anesthesia-related indicators and hemodynamics but may cause blurred vision and diplopia.

As just mentioned, our results indicate that preoperative HPCD and postoperative Atr seem to cause blurred vision and diplopia in patients. Since both HPCD and Atr are anticholinergic agents [10, 11], we speculated that the occurrence of blurred vision and diplopia might be related to the action intensity and half-life of the two. PHCD is more selective than Atr and has strong and longer-lasting effects with a half-life of about 10.34 h versus 3 h-4 h of Atr. According to our results, postoperative combined with postoperative Atr may be insufficient to induce intraocular pressure due to the dose and duration of action, while PHCD combined with Atr may block the effect of acetylcholine on sphincter iridis to a greater extent, thereby increasing intraocular pressure and causing blurred vision [12]. Besides, the comparison of postoperative hemodynamic parameters (HR, MAP, and SpO_2_) showed no significant difference between the two groups, suggesting the combination of PHCD and Atr did not affect the safety of GA. In other words, PHCD combined with Atr may be feasible during induction of GA. Therefore, preoperative and postoperative medication should be carefully selected in the induction period of GA. For patients requiring preoperative use of Atr, preoperative Atr should be avoided, while for those who have been administered PHCD preoperatively, Atr should not be used postoperatively.

However, our findings may be limited by the sample size. In the follow-up investigation, we will expand the sample size and establish a validation set to verify the conclusions obtained in the present study.

## 5. Conclusion

To sum up, preoperative PHCD combined with postoperative Atr may cause postoperative blurred vision and increase the risk of diplopia. Considering the possible after-effects of blurred vision and diplopia, we suggest that the combined use of PHCD and Atr should be avoided in clinical practice, or Atr rather than PHCD should be used before surgery, so as to reduce the occurrence of blurred vision, diplopia, and other ARs.

## Figures and Tables

**Table 1:** General information of patients.

	Research group (*n* = 234)	Control group (*n* = 239)	*t*/*χ*^2^	*P*
Gender			0.369	0.546
Male	125 (53.42)	121 (50.63)		
Female	109 (46.58)	118 (49.37)		
Age (year)	53.12 ± 15.97	53.02 ± 16.65	0.081	0.935
Weight (kg)	61.46 ± 11.17	60.02 ± 13.83	1.446	0.149
ASA classification^∗^			1.483	0.477
Grade I	53 (22.65)	62 (25.94)		
Grade II	121 (51.71)	126 (52.72)		
Grade III	60 (25.64)	51 (21.34)		

^∗^Note: ASA: American Society of Anesthesiologists.

**Table 2:** Comparison of anesthesia-related indexes between the two groups, mean ± SD (min).

	Research group (*n* = 234)	Control group (*n* = 239)	*t*	*P*
Anesthesia induction time	4.14 ± 1.19	4.20 ± 1.10	0.5298	0.5965
Pain disappearance time	3.81 ± 0.68	3.72 ± 0.61	1.4120	0.1587
Recovery time	11.47 ± 2.01	11.53 ± 1.71	0.3435	0.7314

Note: research group: preoperative penehyclidine hydrochloride+postoperative atropine; control group: postoperative atropine/preoperative atropine+postoperative atropine.

**Table 3:** Comparison of adverse reactions between the two groups, *n* (%).

	Research group (n=234)	Control group (*n* = 239)	*χ* ^2^	*P*
Blurred vision	25 (10.68)	0 (0.00)	26.96	<0.0001
Pain	82 (35.04)	84 (35.15)	0.0006	0.9811
Nausea	26 (11.11)	31 (12.97)	0.3858	0.5345
Vomiting	7 (2.99)	15 (6.28)	2.877	0.0899

**Table 4:** Comparison of hemodynamics of patents after recovery from anesthesia between the two groups, mean ± SD.

	Research group (*n* = 234)	Control group (*n* = 239)	*t*	*P*
HR (times/min)	85.48 ± 12.16	85.34 ± 10.97	0.1308	0.8960
MAP (mmHg)	83.25 ± 10.01	81.76 ± 11.36	1.5080	0.1323
SpO_2_ (%)	99.63 ± 1.31	99.64 ± 0.88	0.1721	0.8634

Note: HR: heart rate; MAP: mean arterial pressure; SpO_2_: oxygen saturation.

## Data Availability

The labeled dataset used to support the findings of this study is available from the corresponding author upon request.
